# Corrigendum to “Enhancing the nutritional value of sweet bell pepper through moderate NaCl salinity” [Heliyon Volume 9, Issue 12, December 2023, Article e22439]

**DOI:** 10.1016/j.heliyon.2025.e43374

**Published:** 2025-05-08

**Authors:** F. Marra, A. Maffia, F. Canino, B. Petrovicova, C. Mallamaci, Mt Russo, M. Iftikhar Hussain, A. Muscolo

**Affiliations:** a Department of AGRARIA, “Mediterranea” University, Feo di Vito, 89122, Reggio Calabria, Italy; b Department of Plant Biology & Soil Science, Universidad de Vigo, Campus Lagoas Marcosende, 36310, Vigo, Spain

In this article, reference [14] was incorrectly cited in the Introduction section, which has been retracted.

*[14] M. Butt, A. Sattar, T. Abbas, R. Hussain, M. Ijaz, A. Sher, Morpho-physiological and biochemical attributes of Chili (Capsicum annum* L.*) genotypes grown under varying salinity levels, PLoS One 16 (11de Assis de Oliveira) (2021), e0257893, https://doi.org/10.1371/journal.pone.0257893*.

This reference should be removed from the reference list and the article.

The authors apologize for the errors.

## Declaration of competing interest

The authors declare that they have no known competing financial interests or personal relationships that could have appeared to influence the work reported in this paper.

